# De novo transcriptome analysis of white teak (*Gmelina arborea* Roxb) wood reveals critical genes involved in xylem development and secondary metabolism

**DOI:** 10.1186/s12864-021-07777-x

**Published:** 2021-07-02

**Authors:** Mary Luz Yaya Lancheros, Krishan Mohan Rai, Vimal Kumar Balasubramanian, Lavanya Dampanaboina, Venugopal Mendu, Wilson Terán

**Affiliations:** 1 Department of Biology, Pontificia Universidad Javeriana, Carrera 7 N° 43-82, Bogotá, 110231 Colombia; 2 Department of Plant and Soil Sciences, Fiber and Biopolymer Research Institute, Texas Tech University, Lubbock, TX 79409 USA; 3 Department of Plant and Microbial Biology, College of Biological Sciences, University of Minnesota, Minneapolis, MN USA; 4 Environmental Molecular Sciences Laboratory, Pacific Northwest National Laboratory, Richland, WA USA

**Keywords:** RNA-seq, Xylem, Differential gene expression, Wood development

## Abstract

**Background:**

*Gmelina arborea* Roxb is a fast-growing tree species of commercial importance for tropical countries due to multiple industrial uses of its wood. Wood is primarily composed of thick secondary cell walls of xylem cells which imparts the strength to the wood. Identification of the genes involved in the secondary cell wall biosynthesis as well as their cognate regulators is crucial to understand how the production of wood occurs and serves as a starting point for developing breeding strategies to produce varieties with improved wood quality, better paper pulping or new potential uses such as biofuel production.

In order to gain knowledge on the molecular mechanisms and gene regulation related with wood development in white teak, a de novo sequencing and transcriptome assembly approach was used employing secondary cell wall synthesizing cells from young white teak trees.

**Results:**

For generation of transcriptome**,** RNA-seq reads were assembled into 110,992 transcripts and 49,364 genes were functionally annotated using plant databases; 5071 GO terms and 25,460 SSR markers were identified within xylem transcripts and 10,256 unigenes were assigned to KEGG database in 130 pathways. Among transcription factor families, C2H2, C3H, bLHLH and MYB were the most represented in xylem. Differential gene expression analysis using leaves as a reference was carried out and a total of 20,954 differentially expressed genes were identified including monolignol biosynthetic pathway genes. The differential expression of selected genes (*4CL*, *COMT*, *CCoAOMT*, *CCR* and *NST1*) was validated using qPCR.

**Conclusions:**

We report the very first de novo transcriptome of xylem-related genes in this tropical timber species of commercial importance and constitutes a valuable extension of the publicly available transcriptomic resource aimed at fostering both basic and breeding studies.

**Supplementary Information:**

The online version contains supplementary material available at 10.1186/s12864-021-07777-x.

## Background

Tree wood is considered as a sustainable alternative source for biofuel production [1] in addition to its current use in paper and pulp industries. Manipulation of woody biomass for various applications requires extensive knowledge of the pathways involved in the wood production [2, 3]. In rice, for instance, edition of a CAD (cinnamyl alcohol dehydrogenase) encoding gene using CRISPR-CAS (Clustered Regularly Interspaced Short Palindromic Repeats- CRISPR Associated Nuclease) technology, altered cell wall compostion, reducing lignin content and increasing both cellulose and hemicellulose, which enhanced significantly the saccharification process [4]. A similar result was achieved in poplar, finding that a reduction in lignin biosynthesis led to an improvement of the biomass quality with higher saccharification efficiency [5]. *Gmelina arborea* Roxb. (white teak, Malay beechwood, Kashmir tree, gamari or yemane) is a fast-growing tree species belonging to the lamiaceae family, with tremendous economic importance in several tropical and subtropical areas of southeastern Asia, Africa and America. Its introduction and excellent adaptation to the American tropics (Costa Rica, Venezuela, Colombia and Guatemala) is due to the traits like fast growth, high biomass production (20–25 m^3^/ha/year), less susceptibility to the local pests and high yields in addition to the versatility of its wood use which allow a faster investment return [6]. Therefore, it is considered as a species of choice for both reforestation programs and agroforestry systems in these areas [6, 7].

White teak has also shown natural tolerance to water stress and resistance to fire, both characteristics of high interest in the context of climate change. This species has been considered as a tree with higher bioenergetics production, generating an average of 265 m^3^ of biomass/ha/year [8]. White teak fruits and seed present interesting potential as sources of oil for biodiesel production whereas its lignocellulosic wastes serves as a source of bioethanol [8–10]. Wood is primarily composed of vascular cambium in the woody plants and is composed mainly by secondary xylem. Xylem allows water transport through the stem as well as the tree branches in addition to providing structural support [11].

Formation of wood xylem cells involves two basic processes occurring simultaneously i.e. formation of the secondary cell-wall and programmed cell death [11]. The secondary cell wall is mainly composed of cellulose, hemicellulose and lignin polymers in various proportions [12]. Cellulose is a linear polymer of beta 1–4 linked glucan units that forms microfibers structures which interacts with complex polymers collectively called hemicelluloses in order to form a reticulated matrix [13]. Lignin is a polyphenolic compound which is hydrophobic in nature filling the spaces between celluloses and hemicellulose fibers and conferring additional mechanical support, rigidity and hydrophobicity [14, 15]. After cellulose, lignin is the second most abundant polymer produced by plants, representing approximately 30% of the organic carbon in the biosphere [16]. Lignin polymers are produced from the hydroxycinnamyl alcohol (monolignol) pathway derived from phenylpropanoid pathway, which is also a source of other compounds such as flavonoids, coumarins, phytoalexins and lignans that are important for plant defense against biotic stressors and commercial biomolecule production [17, 18]. Lignin plays a significant role in the growth and development of woody species which adds the required strength to grow upright and withstand against the mechanical pressure [15].

Lignin biosynthetic pathway involves eleven enzymes in order to produce three monolignols; *p*-coumaryl alcohol, sinapyl alcohol and coniferyl alcohol [19]. Polymerization of these monolignols produces three types of lignin units, Hydroxyphenyl lignin (H-lignin), Syringyl lignin (S-lignin) and Guaiacyl lignin (G-lignin) and the type of lignin varies based on the species, tissue type and stage of development [12]. The gymnosperm lignin is mainly composed of H and G units, while angiosperms lignin from monocots is composed of H, G and S units whereas in dicots it is composed of G and S units [20, 21].

Various transcription factors have been identified and characterized as key players of wood development, primarily members of NAC and MYB families involved in the regulation of monolignol pathway and lignin polymerization [19, 22, 23]. The NAC family, the transcription factors SND1, NST1, VND6 and VND7 have been recognized as master switches involved in activation of cascade of transcription factors, converging ultimately into secondary xylem formation and lignification [23, 24], . The MYB family transcription factors appears to directly regulate the lignin biosynthetic as well as other cell wall biosynthetic genes. These MYB transcription factors recognize specific DNA sequence motifs on the promoter or regulatory regions of target genes and thereby activating or repressing transcriptional expression [23–25].

The monolignol pathway has been mainly studied in model plant species such as Arabidopsis and poplar [26, 27]. The knowledge generated from these species, has been used to modify tree species such as poplar and eucalyptus in order to reduce the lignin content [28–30]. Although white teak woody biomass presents a high potential for novel uses, lack of knowledge on metabolic and regulatory genes involved in wood development and lignin biosynthesis impairs its use for biofuel applications. A comprehensive knowledge on lignification pathways and its regulation is essential for the improvement of commercially important traits such as wood quality, paper pulping or biofuel production. Therefore, in the present study we have generated de novo xylem transcriptome and analyzed and identified xylem specific metabolic and regulatory genes which serve as target genes for future breeding developments in this species.

## Results

### Generation and annotation of a reference xylem transcriptome

**Fig. 1:**
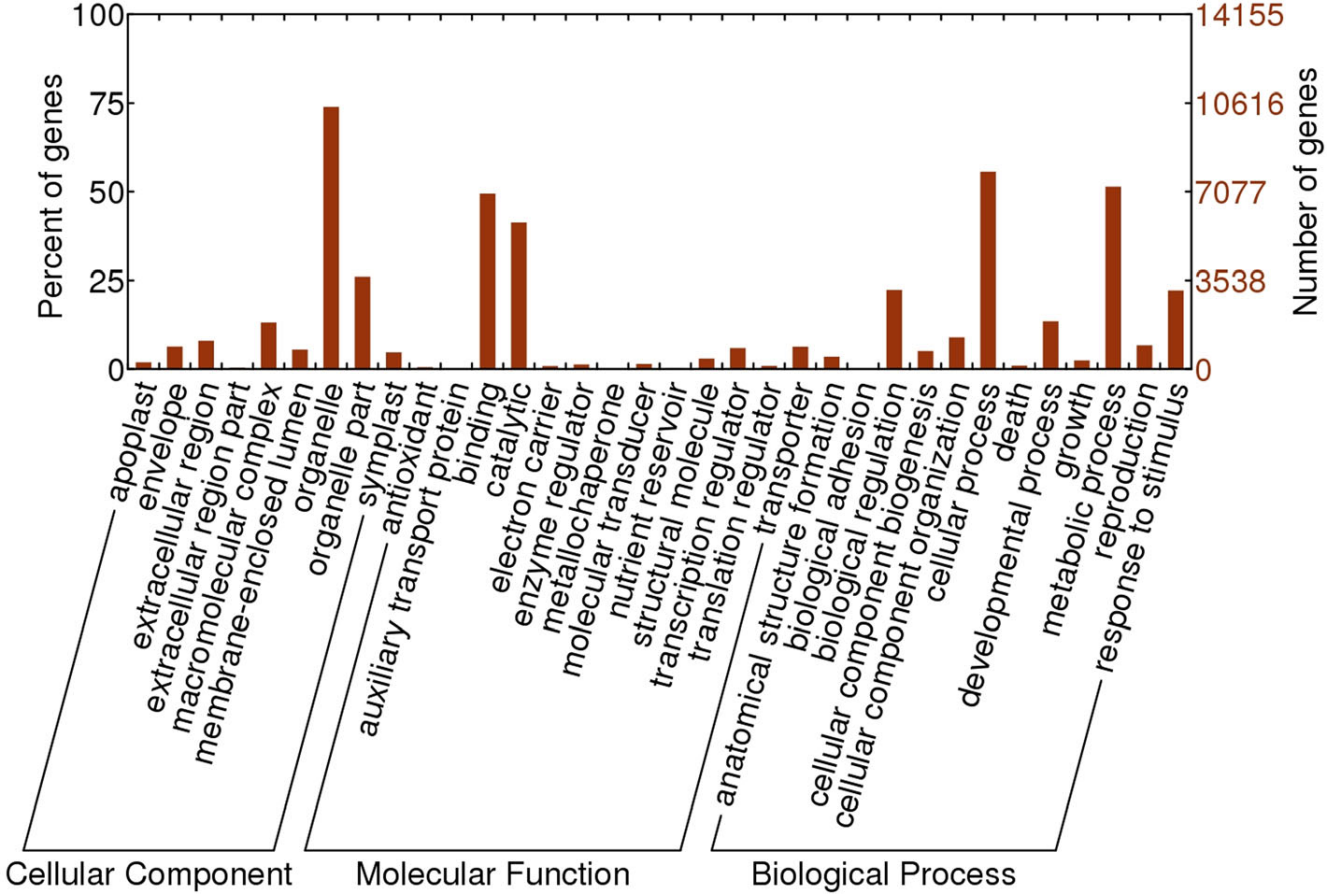
Main GO categories assigned to xylem reference transcriptome of *G. arborea*

RNA-seq of *G. arborea* xylem library resulted in approximately 165 million paired reads. Quality filtration for the low-quality reads (Q < 20) and contaminants such as reads of ribosomal and organellar origin resulted in the removal of total of 18,968 paired sequences. The cleaned reads were assembled using Trinity software to obtain the reference transcriptome with 110,992 transcripts. The assembled transcripts showed a considerably higher N50 value of 1466 bases with the average transcript length of 864 bases (Table 1). Various publicly available tools and databases were used to annotate these assembled *G. arborea* transcripts. A more popular and conventional homology-based annotation with NCBI NR database resulted in 49,364 hits whereas using model plant *Arabidopsis thaliana* TAIR10 protein database resulted in 45,377 hits representing 15,445 unigenes. A higher percentage of transcripts with functional annotation was obtained with HMMER analysis: 64,186 transcripts presented hits with PFAM database. Figure 1 represents the main Gene ontology (GO) categories assigned for 14,155 unigenes. At the level of cellular component, most of the transcripts were located in the category of organelle whereas at the level of molecular function, the binding and catalytic function categories were the most representative. Cellular and metabolic process were among the most significant biological processes, as well as some categories probably related with dynamic activity in xylem tissue like cell biogenesis, and development processes.

**Table 1:** Summary of assembly and annotation metrics of the reference transcriptome obtained from *G. arborea* secondary xylem

**Assembly**	
Total number of sequences obtained	164,737,322
Number of sequences used for the assembly	164,718,354
Number of transcripts obtained post assembly	110,992
N50 value (in bp)	1466
Average contig length (in bp)	864
Putative gene number	81,269
Number of bases assembled	~ 95 M
**Annotation**	
Full length ORFs	17,809 (16%)
Quasi full length ORFs	14,017 (12.6%)
Transcripts with hits in the NCBI NR database (BLASTX)	49,364
Transcripts with hits in TAIR10 (BLASTX)	45,377
Transcripts with hits in *Populus trichocarpa* database	46,795
Transcripts with hits in the NCBI NR base (BLASTX)	45,708
Transcripts with PFAM domains	64,186
Transcripts classified in gene families	48,322
Transcripts with GO terms	39,465
Number of GO terms	5701
Number of KEGG pathways identified	130
Number of genes associated to KEGG pathways	10,256

Using the KEGG (Kyoto encyclopedia of genes and genomes) database, 10,256 genes were assigned to 130 metabolic pathways (Table 1 and 2). Biosynthesis of secondary metabolites, ribosomes and transduction of hormonal signals were the pathways with highest number of associated genes. Phenylpropanoid biosynthesis was also in the top 20 of the most representative pathways (Table 2).

**Table 2:** Top 20 KEGG pathways identified in the *G. arborea* xylem transcriptome

Pathways identified	Number of genes
Metabolic pathways	1841
Biosynthesis of secondary metabolites	1020
Ribosome	346
Transduction of signals of plant hormones	262
Carbon metabolism	256
Aminoacid biosynthesis	251
Protein processing in endoplasmic reticulum	217
starch and sucrose metabolism	194
Spliceosome	189
RNA transport	165
Purine metabolism	156
Plant-pathogen interaction	154
Phenylpropanoid biosynthesis	152
Oxidative phosphorylation	149
Ubiquitin mediated proteolysis	148
Endocytosis	140
Amino sugar and nucleotide sugar metabolism	135
Glycolysis / Gluconeogenesis	113
Pyrimidine metabolism	112
Cysteine and methionine metabolism	112

### Identification of transcription factors, metabolic and regulatory genes involved in the monolignol pathway

**Fig. 2:**
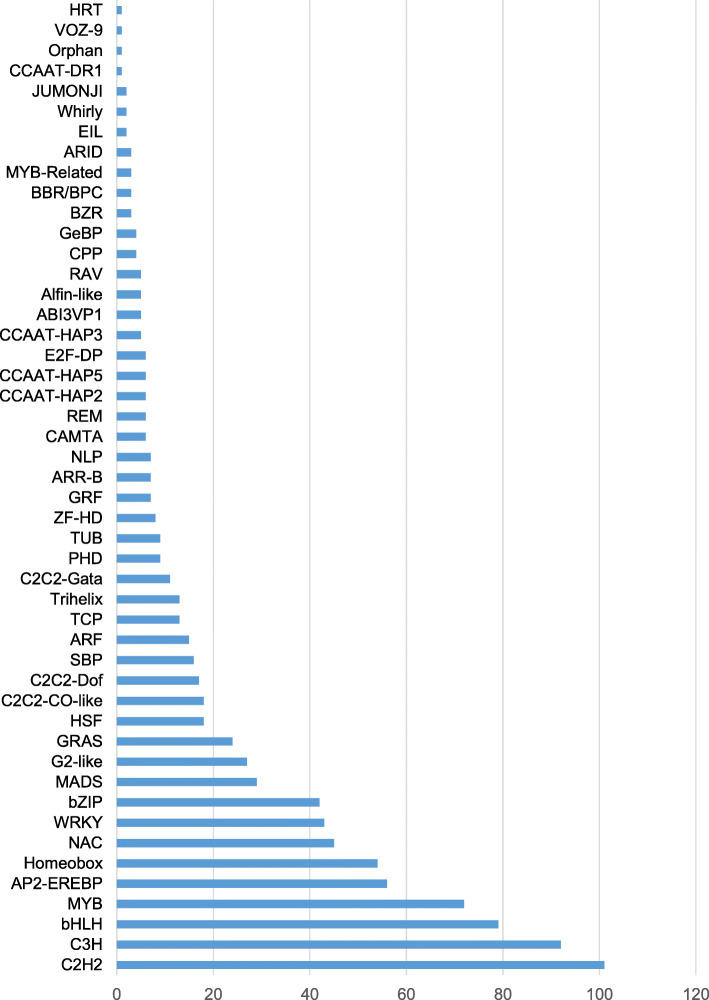
Main families of transcription factors identified in the xylem transcriptome**.** Blue bars indicate the number of transcripts belonging to each family

The main families of transcription factors identified in the reference transcriptome are presented in Fig. 2. 101 unigenes were assigned to *C2H2*, 92 to *C3H,* 79 to *bHLH* and 72 to *MYB* TF families; whereas 240 genes were assigned to the AP2-EREBP (56 genes), Homeobox (54 genes), *NAC* (45 genes), *WRKY* (43 genes) and *bZIP* (42 genes) TF families. Nine biosynthetic genes of the monolignol pathway and transcription factors of different levels of regulation were identified from the reference transcriptome.

**Fig. 3:**
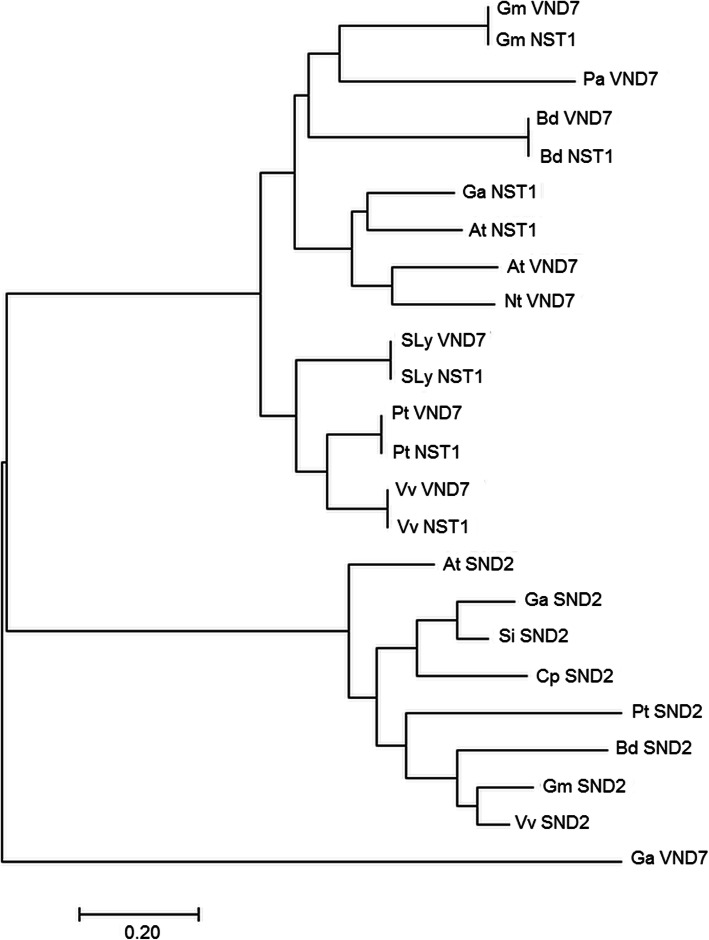
Phylogenetic analysis of *G. arborea* NAC transcription factors: VND7, NST1 and SND2 protein sequences identified from the reference transcriptome of *G. arborea* (Ga) were compared to homologs from other species: At: *Arabidopsis thaliana* (*Q9C8W9*, *Q84WP6*, *O49459*), Bd: *Brachypodium distachyum* (*Bradi1g04150.1.p*, *Bradi1g06970.1.p*, *Bradi1g37898.1.p*), Cp: *Carica papaya* (*XP_021889039*), Gm: *Glycine max* (*XP_006589457.1*, *Glyma.01G046800.1.p*, *Glyma.01G005500.1.p*), Nt: *Nicotiana tabacum* (*XP_016440678.1*), Pa: *Picea abis* (*MA_101849g0010*), Pt: *Populus trichocarpa* (*XP_024447115.1*, *Potri.001G061200.1*, *Potri.001G343800.1*), Si: *Sesamum indicum* (*XP_011096365*), Sly: *Solanum lycopersicum* (*Solyc01g009860.2.1*, *Solyc01g102740.2.1*), Vv: *Vitis vinifera* (*GSVIVT01000940001*, *XP_002267383*, *GSVIVT01015274001*). The clustering method used for dendrogram construction was neighbor-joining. Line length indicates the evolutionary distance. Uniprot, NCBI protein, TAIR and PlantTFDB accession IDs are shown in parenthesis. In the case of *Picea abis*, accession was obtained from iTAK plant transcription factor database

Among the NAC transcription factors, putative orthologs of Arabidopsis *VND7, SND2* and *NST1*, reported as “master” regulators, were identified. In the case of MYB transcription factors, *MYB46* and *MYB83,* which were classified as regulators of second level, and *MYB20*, *MYB69* and *MYB85* which are directly related with the activation of monolignol biosynthetic genes, were identified. Other important transcription factor encoding genes were found like *MYB7*, *MYB4, MYB32* and *KNAT7*, all reported as negative regulators, or *BES1* a specific activator of the synthesis of celluloses (Table 3). In order to clarify the relation and identity of NAC transcription factors identified as *VND7*, *SND2* and *NST1*, a phylogenetic analysis using possible orthologs from other species was performed (Fig. 3).

**Table 3:** Genes related with lignin biosynthesis and its regulation, identified in the xylem reference transcriptome

Group	Identified genes
Monolignol pathway genes	Phenylalanine ammonia-lyase (*PAL)* [EC:4.3.1.24]
	Cinnamyl alcohol dehydrogenase *(CAD)* [EC:1.1.1.195]
	Ferulate 5-hydroxylase *(F5H)* [EC:1.14.-.-]
	Hydroxycinnamoyl-CoA reductase *(CCR)* [EC:1.2.1.44]
	Caffeic acid O-methyltransferase *(COMT)* [EC:2.1.1.68]
	4-coumarate-CoA ligase *(4CL)* [EC:6.2.1.12]
	p-hydro-xycinnamoyl-CoA *(HCT)* [EC:2.3.1.133]
	caffeoyl-CoA O-methyltransferase (*CCoAOMT)* [EC:2.1.1.104]
	p-coumarate 3-hydroxylase *(C3’H)* [EC:1.14.13.36]
MYB transcription factors	*MYB46*
	*MYB61*
	*MYB83*
	*MYB103*
	*MYB4*
	*MYB7*
	*MYB32*
	*MYB52*
	*MYB20*
	*MYB63*
	*MYB69*
	*MYB85*
NAC transcription factors	*SND2*
	*VND7*
	*NST1*
BES1/BZR1 transcription factors	*BES1*
KNOX transcription factors	*KNAT7*

In dendrogram (Fig. 3), the transcription factor SND2 of white teak was related to orthologs from other plant species, while NST1 presented a closer phylogenetic relationship with the NST1 transcription factor of Arabidopsis. In the case of white teak VND7 transcription factor, it was least related with the corresponding orthologs from other species.

### Identification of single sequence repeats (SSRs) markers

A total of 25,460 exonic SSR markers were identified with 2–5 nucleotides repeat motifs. Among them, the most predominant repetitions were dinucleotides (DNRs, 20,634) and trinucleotides (TNRs, 4463) (Supplementary Table 2). In the case of DNRs, AT and AG were the most abundant motifs (33%) followed by GA and TA (29%). Among TNRs, GAA (4.9%), AAG (4.7%), TTC (4.0%) and CCG (3.7%) were the most abundant motifs.

### Differential expression analysis

With the goal to perform the differential expression analysis between xylem and leaves, we first generated a unique combined transcriptome using the reads from both tissues, since no reference genome sequence is available for *G. arborea*. A total of 196,317,195 sequences were obtained from leaves; after removal of contaminants and low-quality sequences (about 50 millions of reads), 147,130,884 sequences were obtained. For generation of combined transcriptome, the sequences obtained from leaves were fused with the sequences obtained from xylem. The mapping of reads against this transcriptome indicated an average alignment percentage of 95%, which is indicative of a good representability of expressed transcripts in the transcriptome. Metrics related with the assembly and annotation of this transcriptome are shown in supplementary Table 1.

**Fig. 4:**
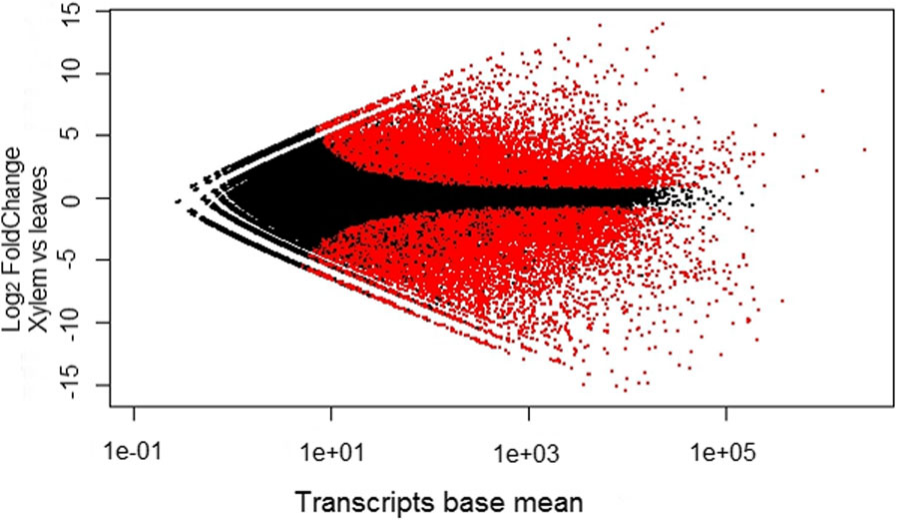
Distribution of differentially expressed transcripts (DEG) with a p-value < 0.05. DEG are shown in red and the non-DEG are shown in black

Using this unique transcriptome as reference, the differential expression analysis between leaves and secondary xylem (stem) was performed using leaf tissue as a control. Principal component analysis (PCA) of transcript expression levels revealed a clear differentiation of the samples according to the tissue type (supplementary Fig. 1). Results, also indicated that 38,350 transcripts were differentially expressed (adjusted *p* value < 0.05), out of which 20,964 showed log 2 fold change (Log_2_FC) absolute values higher than 2 as a threshold: 9011 transcripts showed an induction pattern whereas 11,953 were repressed in xylem compared to leaf tissue (Fig. 4). Main functional categories of DEGs are shown in supplementary Fig. 2.

**Fig. 5:**
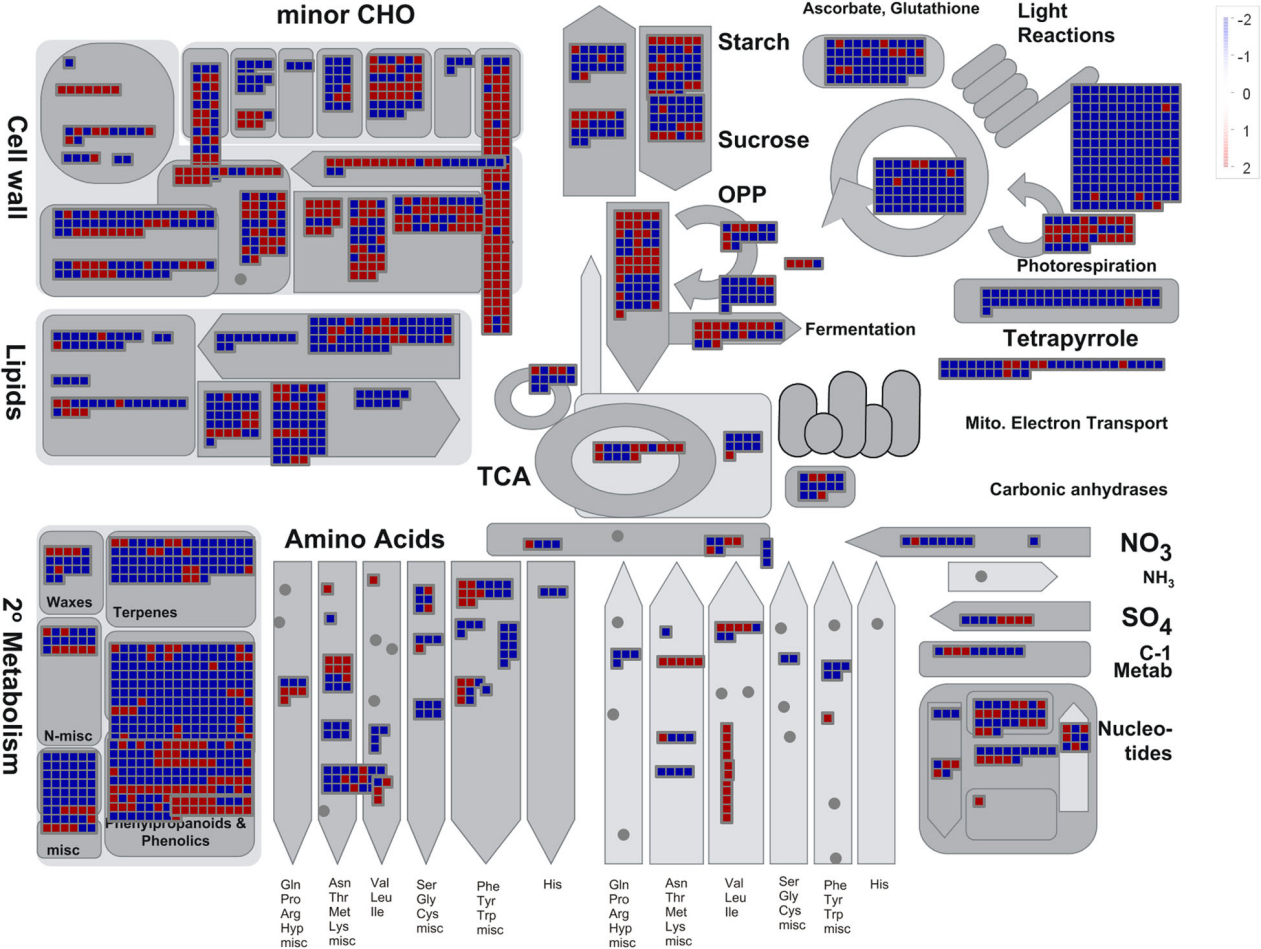
Differential expression between leaf and stem according to the main metabolic processes in which they are involved. The logarithm of changes of expression for each transcript is represented in red color (induction in stem, Log_2_FC ≥ 2) and blue (repression in xylem, induction in leaf, Log_2_FC ≤ -2). Analysis was performed using the MapMan visualization software [93]

To identify overall changes in xylem metabolic pathways encoded by these DEGs, the Mapman tool was used, using the same Log_2_FC thresholds values (|Log_2_FC| ≥ 2). Figure 5 presents a general outlook of induction and repression patterns of transcripts involved in main primary and secondary cell metabolism.

**Fig. 6:**
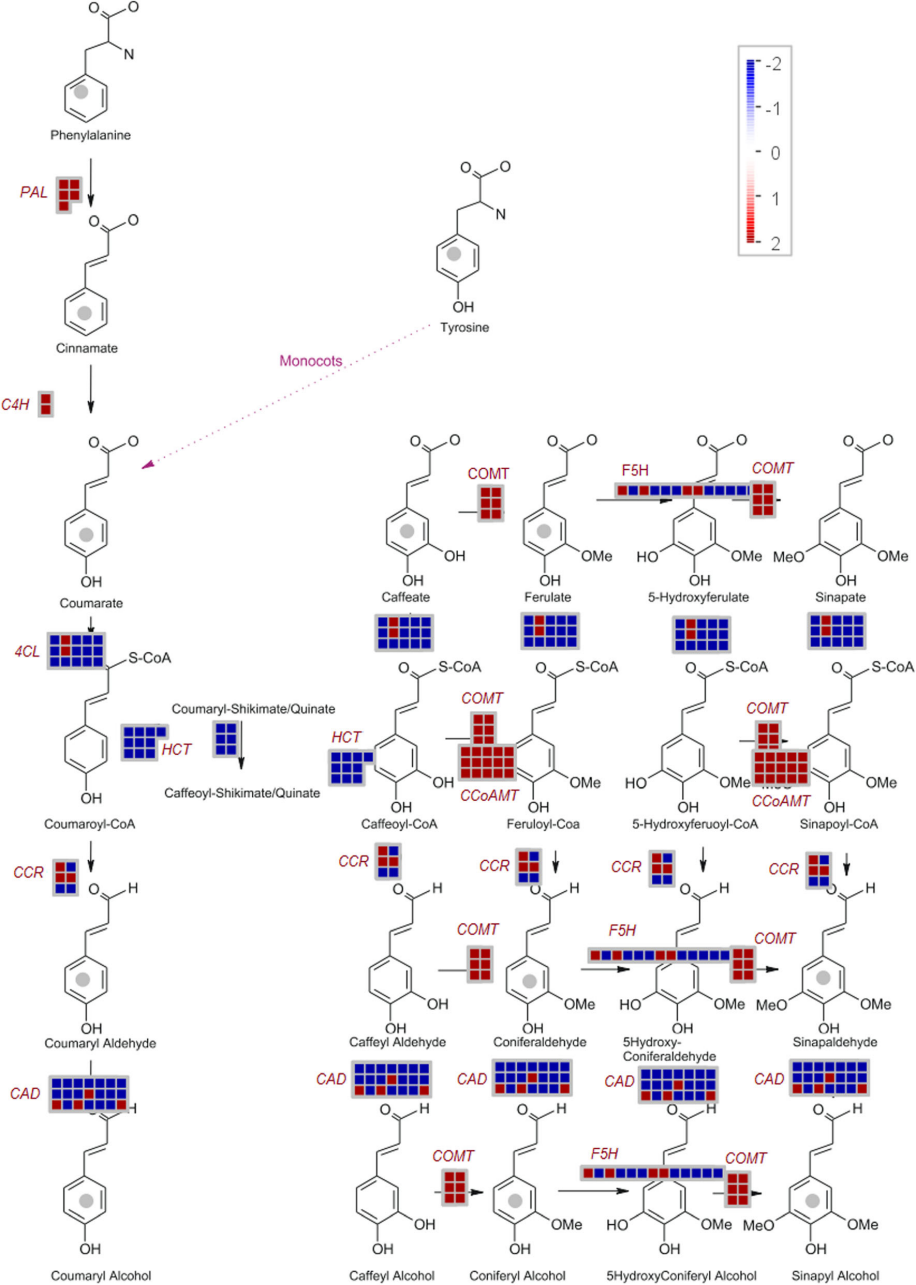
Differential expression of genes of the monolignol pathway, according to the logarithm of fold change (Log_2_FC). Transcripts corresponding to each gene are represented in squares. In red are represented the Log2FC values ≥2 (induction in xylem) and in blue the Log_2_FC values ≤-2 (repressed in xylem). Pathway analysis was performed using the MapMan visualization software [93]

As expected, genes involved in photosynthetic light reactions were clearly repressed in xylem compared to leaves, whereas those related to respiration were induced. Accordingly, genes related to cell wall synthesis tend to show an induction pattern in stem compared to leaves. Analysis of nine genes of the monolignol pathway showed a clear differential expression between leaves and xylem (Fig. 6). A general pattern of higher expression was identified for the *PAL*, *C4H*, *COMT* and *CCoAOMT* genes in xylem, while the *HCT* gene was repressed compared to leaves. In the case of *4CL*, *F5H*, *CCR* and *CAD* different transcripts (associated in various cases with possible splicing isoforms) of the same gene presented a higher expression in one or other tissue.

**Fig. 7:**
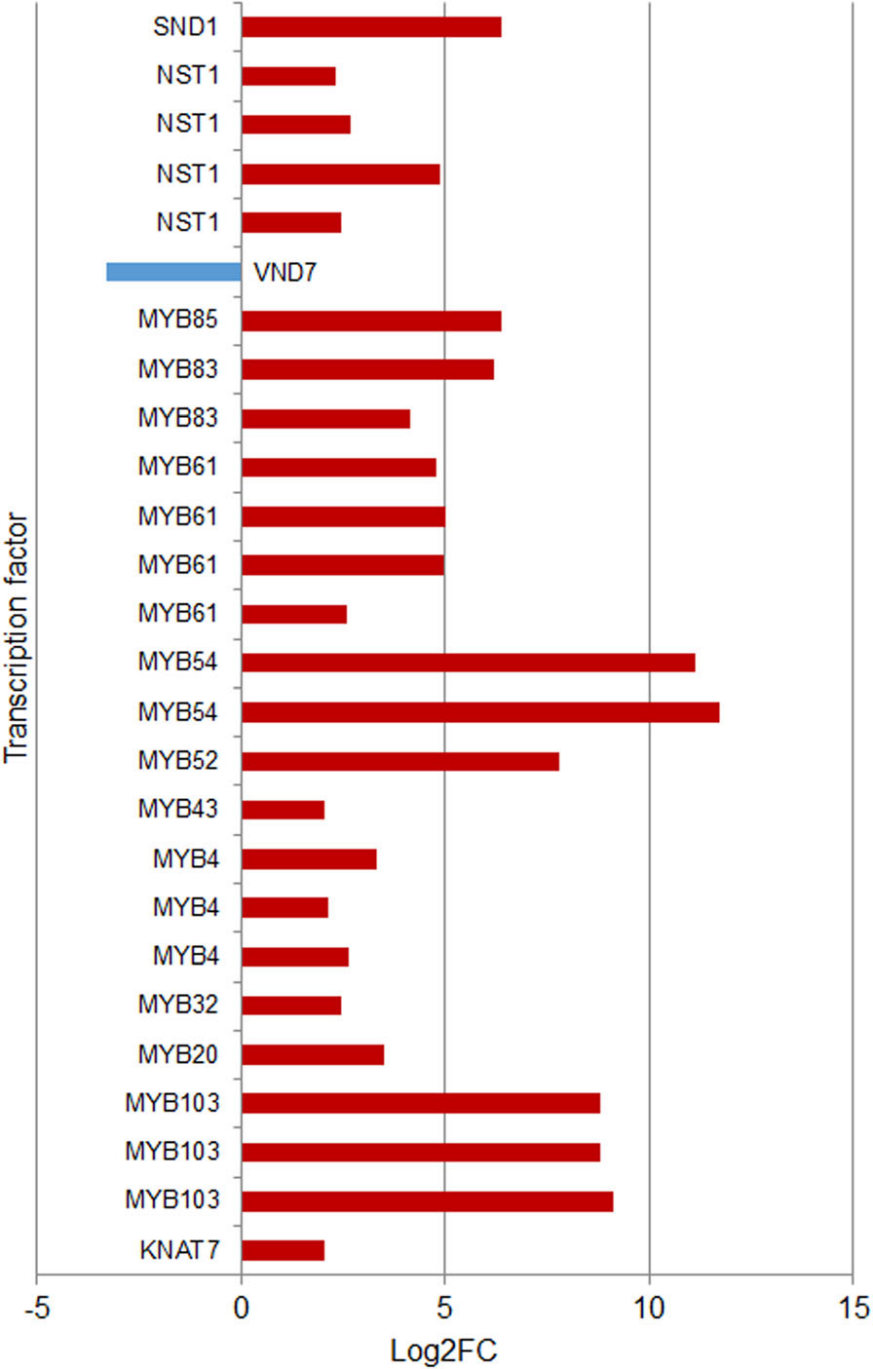
Differential expression of transcript isoforms encoding transcription factors involved in the regulation of the monolignol pathway. Red color represents Log_2_FC values ≥2 (induction in xylem) and blue color Log_2_FC values ≤-2 (repressed in xylem, induction in leaf)

**Fig. 8:**
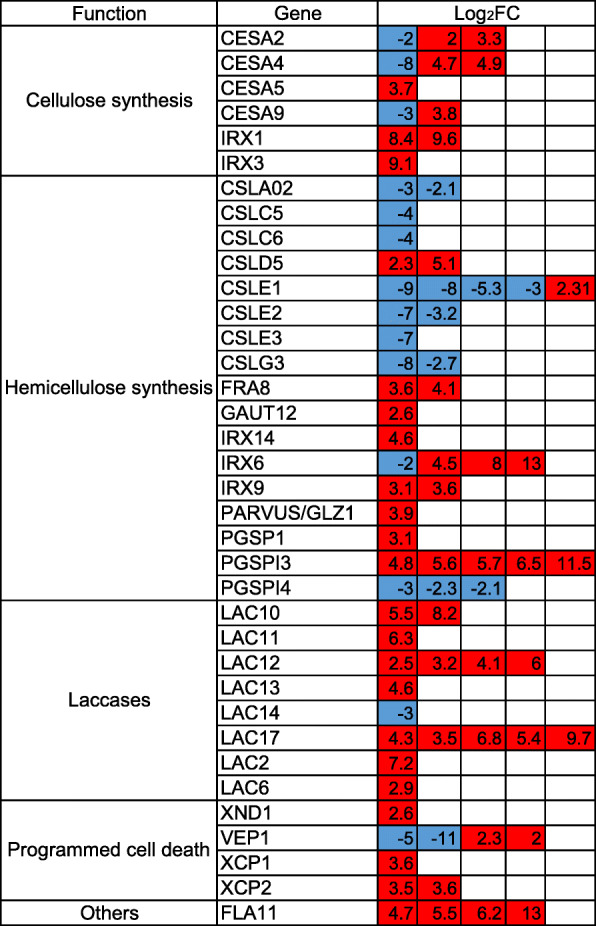
Genes related to the synthesis of other elements of the secondary cell wall with differential gene expression between stem and leaf. Red color represents Log_2_FC values ≥2 (induction in xylem), blue colors Log_2_FC values ≤ - 2 (repressed in xylem). Log_2_FC values of all transcript isoforms of the same gene are presented

Additionally, transcripts encoding transcription factors belonging to MYB, NAC and homeobox families, were differentially expressed (Fig. 7). A clear induction of transcripts annotated as members of MYB family was observed in xylem. In the case of NAC family, several transcripts encoding NST1 transcription factor, were induced in xylem whereas one *VND7* homolog showed a repression pattern in xylem. Finally, *KNAT7*, a member of the homeobox family, was also induced in xylem tissue. Other genes involved in the development of secondary cell wall also showed differences between leaves and xylem (Fig. 8). These genes were further classified into five groups based on their function: cellulose synthesis, hemicellulose synthesis, laccases, programed cell death and others.

### Identification of paralogs and their respective splice variants of genes of monolignol pathway

Genes of monolignol pathway contain several variants or paralogs, which may be involved in the same function or have different functions. The reference transcriptome and the differential expression analysis allowed the identification of these paralogs and their possible splicing isoforms for some of the monolignol pathway genes. In the case of *PAL*, two possible paralogs *PAL1* and *PAL4* were identified and both generated different splicing isoforms, all of them upregulated in stem. In the case of *CAD,* possible orthologs of *CAD9* and *CAD3* were identified; the putative *CAD9* paralog was expressed in both tissues, whereas the *CAD3* was expressed only in stem. Additionally, other two genes, previously not reported, showed a contrasting pattern of expression between tissue: for *4CL,* two transcripts *4CL1* and *4CL2* were identified as possible variants; the last one was induced in leaf, while *4CL1* was mainly induced in stem. Similarly, *CCoAOMT* presented two possible variants *CCoAOMT1* and *CCoAOMT2.* No gene or transcript variants were detected for *C4H*, *COMT*, *F5H*, *CCR*, and *HCT,* as a single transcript was identified.

### Phylogenetic analysis

**Fig. 9:**
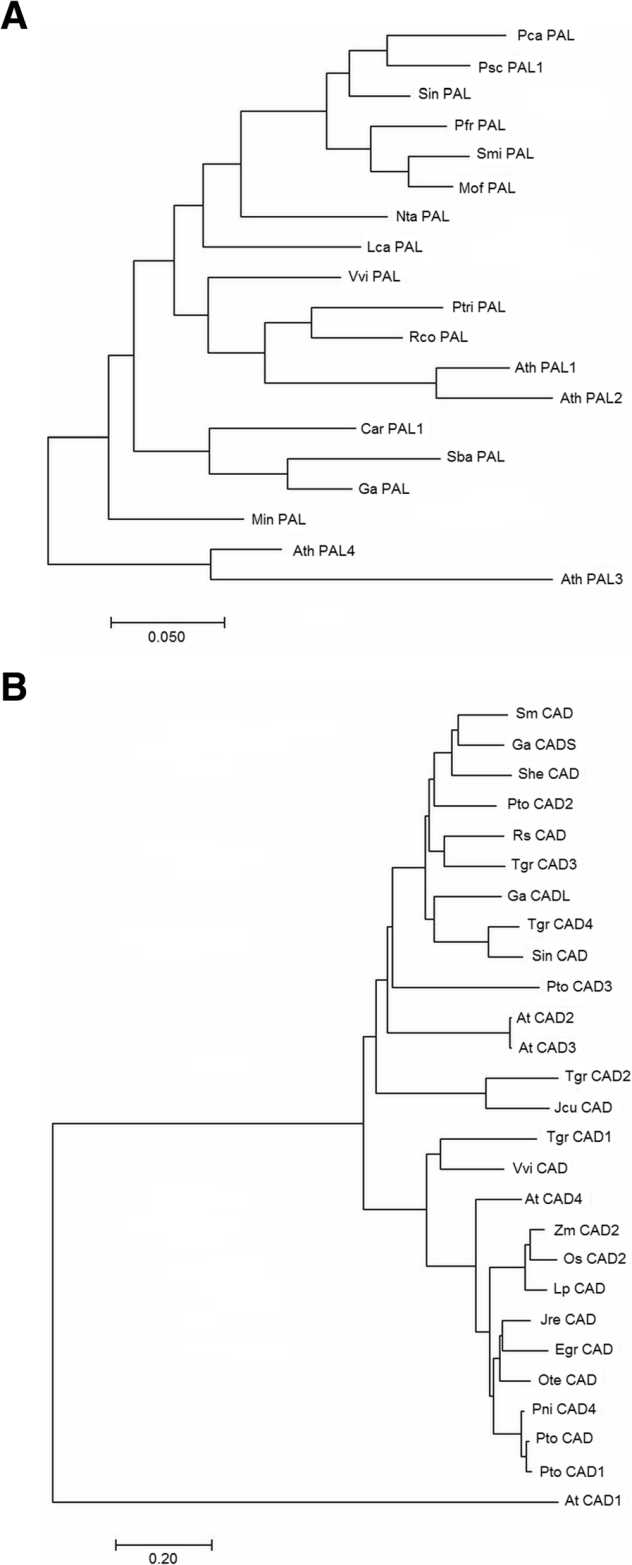
Phylogenetic analysis of *G. arborea* PAL (**A**) and CAD (**B**) proteins. Protein sequences of PAL and CAD enzymes obtained from *G. arborea* full length cognate transcripts were compared to homologous sequences belonging to other plant species. Dendrograms were constructed using the neighbor-joining clustering method. Line length indicates the evolutionary distance. In addition to *G. arborea* (Ga) putative PAL1 sequence, other protein sequences used in PAL phylogenetic analysis were: *Ath: Arabidopsis thaliana,* with four paralogs of PAL included in the analysis, *AthPAL1 (P35510)*, *AthPAL2* (*OAP06573*), *AthPAL*3 (*OAO94639*) and *AthPAL4* (*OAP02490.1*). *Car: Coffea arabica (AEL21616), Lca: Lonicera caerulea (ALU09327), Nta: Nicotiana tabacum (NP_001312352.1), Min*: *Mangifera indica (AIY24975.1), Mof: Melissa officinalis (CBJ23826.1), Pfr: Perilla frutescens (AEZ67457.1)*, *Psc: Plectranthus scutellarioides (AFZ94859.1), Pca: Pogostemon cablin (AJO53272.1), Ptri: Populus trichocarpa (P45730), Rco: Ricinus communis (AGY49231.1), Smi: Salvia miltiorrhiza (ABD73282), Sba: Scutellaria baicalensis (ADN32766.1), Sin: Sesamum indicum (XP_011094662), Vvi: Vitis vinifera (ABM67591),* Protein sequences used in CAD phylogenetic analysis, included two possible variants of *Gmelina arborea (Ga),* the first one induced in stem (CADS, putative CAD3) and the second one induced in leaves (CADL). Other CAD protein sequences used were: *Ath: Arabidopsis thaliana* CAD1 *(OAP16446.1)* and CAD2 *(NP_179765), Egr: Eucalyptus grandis (XP_010024064.1)*, *Jcu: Jatropha curcas (XP_012086572.1), Jre: Juglans regia (XP_018827699.1), Lp: Lolium perenne (AAB70908), Ote: Ocimum tenuiflorum (ADO16245.1), Os: Oryza sativa (Q6ZHS4), Pni: Populus nigra (AFR37935.1), Pto: Populus tomentosa (AAR83343.1), Rs: Rauvolfia serpentine (ALW82980.1), Sm: Salvia miltiorrhiza (ADN78309.1), Sin: Sesamum indicum (XP_011097452.1), She*: *Sinopodophyllum hexandrum (AEA36767.1), Tgr: Tectona grandis (ANG60951.1, ANG60952.1, ANG60953.1, ANG60954.1), Vvi: Vitis vinifera (RVW57228.1), Zm: Zea mays (NP_001105654).* Different CAD members were included for some species. Accession IDs from protein NCBI database are shown in parenthesis

In order to determine the phylogenetic relations of some genes of the monolignol pathway identified in white teak with homologous sequences reported for different species, a dendrogram was generated using the protein sequences obtained from *G. arborea PAL* and *CAD* genes with full length ORFs. These genes are the first (*PAL*) and the last (*CAD*) ones to be involved in the monolignol pathway and are key players for the lignin biosynthesis. In the case of *PAL,* one variant induced in stem (putative *PAL1*) was selected, while for *CAD,* two variants were included: one upregulated in stem (called *CADS* and identified as putative *CAD3*) and another one upregulated in leaf (called *CADL*) (Fig. 9A and B).

In the case of PAL, white teak protein formed a single cluster with another possible ortholog of a Lamiaceae family member, *Scutellaria baicalensi,* and also with *PAL1* of *Coffea arabica* (Rubiaceae). For CAD protein, the two evaluated white teak members appeared in different but closely located clusters where CADS was most related with CAD of *S*a*lvia miltiorrhiza* and *Sinopodophyllum hexandrum*, while CADL was most related with CAD of *Sesamum indicum* and CAD4 of *Tectona grandis* (teak), two species belonging to lamiales order . CAD1 and CAD4 from *T. grandis* (Lamiaceae), a species closely related to *G. arborea*, were located in distant clusters, indicating a high degree of divergence amongst homologous members of this protein family.

### Differential expression validation using quantitative reverse transcription PCR (RT- qPCR)

**Fig. 10:**
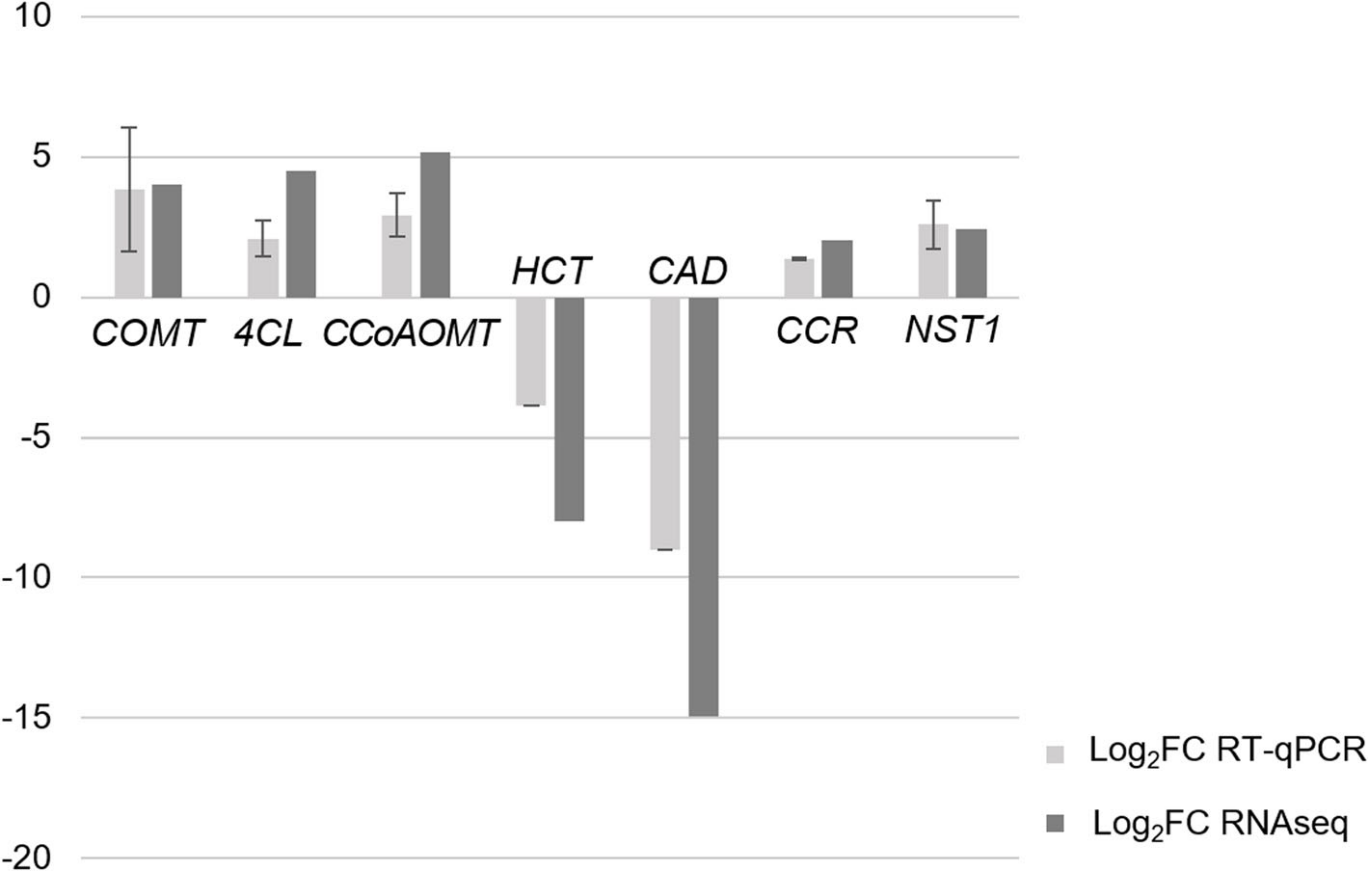
RT-qPCR differential expression validation of a selection of seven *G. arborea* genes. Bars indicate log2FC of xylem expression compared with leaf expression: black bars, mean log_2_FC values obtained from RT-qPCR assays; gray bars, mean log_2_FC values obtained from RNA-seq data

In order to validate the patterns of differential expression observed, a total of 12 genes (10 upregulated and 2 downregulated) were selected for qPCR validation: seven from metabolic genes of the monolignol pathway, two from regulatory genes (transcription factors) and three genes related with synthesis of celluloses and hemicelluloses. For each case, the genes were selected based on the Log_2_FC values obtained previously. Comparing the values between the fold change observed in RT-qPCR and the fold change of gene expression obtained by RNA-seq, a concordance was found between the values for the *COMT*, *CCR* and *NST1* genes. A similar trend in the expression pattern was found for *CCoAOMT*, *4CL*, *HCT* and *CAD* genes (induced in leaf) (Fig. 10) however, no concordance between Log_2_FC values was found for the *MYB85*, *PAL*, *CESA*, *FRA8* and *PGSIP3* genes (data not included). Correlation analysis between the values of Log_2_FC of genes with concordant patterns indicated a moderate general correlation coefficient of 0.50.

## Discussion

In Colombia, white teak plantations are located mainly in the dry tropical Caribbean zone area, characterized by the presence of a bimodal rainfall pattern, in which the plants are frequently subjected to drought periods that can affect the establishment of new plantations and yields [31]. During water stress conditions, it is common to find that the wood lignification patterns are also modified; these modifications have been related with morphological changes in structures like vessels, necessary for an adequate hydraulic conductivity [32]. However, the molecular mechanisms involved in this type of responses are not very clear yet; therefore, it is important to bring knowledge about this type of mechanisms, especially in timber species of high importance, like white teak, whose plantations are frequently under stress conditions. There are only a few species such as *Eucalyptus sp* [33], *Populus sp* [34] and *Pinus radiata* [35] with reported transcriptomic data from xylem, probably due to the difficulty in tissue collection. Further, for tropical timber non-model species, genomic information is still scarce except for some species like acacia [36] and teak [37–39]. Hence, this pioneering study provides information at genomic level associated with development of wood in a non-model tropical species like *G. arborea* Roxb.

The xylem transcriptome contains 110,992 transcripts, up to 60% of these could be annotated using different annotation methods (GO, protein domains, BLASTX, KEGG), and also generated a high percentage of transcripts with full length ORFs (16%) and quasi full length ORFs (12,6%). GO annotation revealed binding and catalysis as main enriched molecular functions. In the binding category, genes related to transcription factors predominated, indicating that this function is critical for the development of white teak’s xylem, while in the catalysis GO category, the importance of different metabolic processes ocurring in this tissue is reflected. One of the most represented category in KEGG pathway was the phenylpropanoid pathway which gives rise to secondary metabolites that are important for different biological processes like pigmentation, UV protection, or responses to pathogens [17]. Additionally, this pathway also produces the monolignols, which are the components of the lignin polymers. Therefore, the results obtained indicate, as expected, a high activity for the pathways involved in the formation of lignin in the developing wood. The de novo transcriptome assembly approach allowed to identify and annotate nine of the ten metabolic genes of the monolignol pathway, which are involved not only in lignin formation but also in other biological processes [40]. Further functional characterization of these individual genes and their variants will provide more information on their biological importance.

### Analysis and identification of exonic SSR markers

Identification of genetic polymorphisms from transcriptomic data, like SSRs markers, is also relevant for a non-model species as it can be used in future studies for associating genotype/phenotype oriented towards germplasm bank characterization and breeding processes. The analysis of SSRs markers in the white teak xylem-transcriptome indicated a predominance of the dinucleotides AT and AG, which is in accordance with studies in different dicot and gymnosperms species [41]. The xylem transcriptome of white teak showed the GAA/AGG (9.9%) repetitions and TTC/CCG (7.7%) as the most common SSRs. The AGG motif has been reported as highly frequent in monocot species [42], while GAA has been identified mainly in regulatory regions in Arabidopsis [43]. It has been reported that trinucleotides are less common than dinucleotides; however, their presence in coding regions, may be related to functional polymorphisms while maintaining intact open reading frames.

### Analysis of wood and secondary cell wall developmental genes

In order to identify genes more specifically related with the wood development in white teak, the transcriptional profiles of growing trunks (secondary xylem) and leaves from young trees were contrasted. Differential expression analysis evidenced that, in the case of leaves, various transcription factors, predominantly upregulated, were related to leaf development and photomorphogenesis processes such as *KAN* family members that have been related to the abaxial identity [44], *MYB-like* related to foliar senescence [45] and *ELF3* related to development and flowering [46]. In the case of xylem, the significant activation of genes related to development of secondary cell wall was evidenced, which is in accordance with the developmental stage or maturity of the sampled trees. Analysis of the transcription factors involved in the regulation of secondary cell wall biosynthesis showed that *C2H2* and *C3H*, which are involved in the hormonal signal transduction process and different processes of development and response to stress in plants were the most abundant [47, 48]. Further, the *MYB* and *NAC* families, which are involved in different biological processes like response to biotic and abiotic stress, cell cycle control, amongst others [49, 50] were highly represented. These transcription factor families act like “master” regulators at different levels in the secondary cell wall development. Particularly, members of the *NAC* family of transcription factors such as *SND2*, *VND7* and *NST1* act as activators in the third and second level of the regulatory network [51]. The MYB transcription factors act as activators and repressors of secondary cell wall biosynthetic genes [52, 53]. Interestingly, members of all the above families were represented and upregulated in the stem xylem of white teak.

The secondary cell wall master regulator NAC transcription factors showed a general significant pattern of induction in stems was observed for *NST1* and *SND2* genes, whereas the transcript annotated as *VND7* was downregulated. NST1 is involved mainly in the regulation of development of xylem fibers as has been reported for different species like Arabidopsis and Poplar [15, 54]. In the case of VND7, although, it has been mainly related to regulation processes in the secondary cell wall formation of vessels [53], its low expression in stem could indicate that its role may be dynamic. This is in agreement with the observation by Mitsuda et al. [54], who affirm that although NST and VND are similar in their functions, there are some differences in the way in which they act during the formation of the secondary cell wall, being the NST factors more consistant in their expression and VNDs more variable. However, it is necessary to validate the identity of this transcription factor, because the phylogenetic analysis was inconclusive. The direct downstream targets of NST1, MYB family of transcription factors such as MYB46, MYB61, MYB83 and MYB103 were significantly induced in stem. These transcription factors are involved in regulating other factors such as MYB52 and SND2, related with the direct upregulation of secondary cell wall biosynthetic genes [52], as well as MYB family belonging repressors, like MYB4, MYB7 and MYB32. Genes encoding other downstream acting MYB factors, directly related with the regulation of the lignin synthesis, were upregulated in stem, such as *MYB20*, *MYB63*, *MYB69* and *MYB85*. Interestingly, the repressor genes *KNAT7* and *MYB4* were also found to be significantly induced in stem, which suggests the presence of negative feedback control loops induced along the development processes of *G. arborea* secondary cell wall.

### Analysis of lignin biosynthetic genes

Specifically, the phenylpropanoids pathway showed a clear pattern of upregulation in xylem compared to leaves, as exemplified by *PAL*, *C4H*, *COMT* and *CCoAOMT* genes (Fig. 6). However, some variants of biosynthetic genes behave differently. Homologous genes or transcript variants contribute to functional redundancy as well as phenotypic plasticity, where specialization may take place, giving rise to organ or environmental dependent expression. In the case of the *PAL* gene, four variants have been reported in Arabidopsis (*PAL1*, *PAL2*, *PAL3* and *PAL4*) [55], all of them with high importance in the process of lignin biosynthesis. Whereas in tobacco, it has been reported that *PAL2* is more related to processes of development of leaves and flowers as well as pollen viability [40]. In our transcriptomic profiling, unique white teak’s variants for *COMT* and *C4H* were identified and both were significantly upregulated in stem, whereas *F5H* and *4CL* were expressed in both tissues, which does not exclude the possible presence of other variants or multi-functionality of a same variant in other tissues or developmental process.

In the case of CAD enzyme, which catalyzes the last step of the biosynthesis of monolignols for the formation of the alcoholic forms, 9 different members have been reported in Arabidopsis and 12 in rice*,* some of them with different patterns of expression among different types of tissues [40, 56]. In the xylem of white teak 4 possible variants of *CAD* gene were identified, among which *CAD3* showed a predominant expression in stem and *CAD9* was equally expressed in both tissues, which could indicate a multifunctional role for this gene. *CAD9* has been related mainly to the lignification processes [57], with a gradual induction pattern during stem developmental stage succession [58], although its expression has also been evidenced in leaves and as part of stress response mechanisms [58–60]. The identity of the other two possible *CAD* members was not determined, however both of them were predominantly expressed in leaves of white teak. Besides, some Arabidopsis variants of *CAD* (i.e. *CAD2* and *CAD3*) are poorly or not expressed during lignification processes, thereby indicating probable different roles in other biological processes [40]. Phylogenetic analysis showed the relationship of two variants of CAD proteins found in white teak,with other possible homologs; the CADs variant (putative CAD3) was tightly related with *S. milthiorrhiza* CAD*,* whereas CADL grouped together with CAD4 of *T. grandis* and CAD of *S. indicum*, indicating its possible relation with other members of the lamiales order. However, a more in-depth analysis is necessary to determine the specific identity, ortholog relationship, and biological function of all these members found in white teak.

Differential expression analysis showed that a unique *HCT* gene was significantly upregulated in white teak leaves. According to Besseau et al. [61], under certain conditions, *HCT* may have a key role in the synthesis of flavonoids which may be the case in the leaves of white teak. Xylem expression of HCT, although lower, could be enough to maintain the lignification process.

#### Biosynthetic genes involved in non-lignin components of secondary cell wall

Development of the xylem cells requires coordinated synthesis of the different elements constituting the secondary cell wall and programmed cell death. Some of the genes involved in these processes showed highly specific expression patterns. This is the case of *IRX* (Irregular xylem) genes, whose mutations affect the phenotypic development at the level of xylem cells [62] as well as *PGSIP* (plant glycogenin-like starch initiation proteins) genes, also known as *Gux,* that constitute a group of genes involved in xylan synthesis and whose function has been specifically related with secondary wall formation [63]. The *IRXs* genes are involved in synthesis of celluloses and hemicelluloses: *IRX1*, *IRX3* and *IRX5,* for example, are cellulose synthases (CesA) specifically expressed in secondary cell wall [64]. Interestingly, key protease encoding genes such as *XCP1*, *XCP2* and *VPE*, known to be involved in programmed cell death during xylem development, have been identified amongst xylem upregulated genes [65]. Furthermore, concomitant upregulation of genes encoding transcription factors like VNI2 and XND1, reported as specifically involved in the tight regulation of this process, has also been observed in our transcriptomic profile [11].

On the other hand, specific activation of laccase genes such as *LAC4*, *LAC11*, *LAC17*, *LAC10* and *LAC2*, known to be involved in monolignol polymerization [66–68], may reflect the importance of these enzymes for xylem formation. Finally, upregulation of the *FLA11* gene in xylem is in accordance with previous reports of its induction during the biosynthesis of the secondary cell wall in Arabidopsis and Eucalyptus, where a key role for these fasciclin-like arabinogalactan proteins in cell wall development biomechanics and development has been proposed [64].

Other key genes showed a different pattern of expression like those coding for cellulose synthases (*CesA*), and cellulose synthase-like proteins (*CSL*), for which a significant downregulation in stem was observed. This could be related to fluctuations in the expression of these genes according to the type of cell wall, and the developmental stage. In Arabidopsis for example, expression of *CesA1*, *CesA2*, *CesA3*, *CesA5*, *CesA6* and *CesA9* genes was shown to be related to formation of primary cell wall, rather than secondary cell wall [69]. In rice and *Eucalyptus camaldulensis*, differences in the patterns of expression of some *CesA* were found in different types of tissue,cell wall or development stages [70, 71]. In the case of the *CSL* genes, in white teak most of them presented a predominant expression in leaves. About this, Lerouxel et al. [72], and Muthamilarasan et al. [73], indicate that these proteins have a relevant role in the synthesis of polysaccharides that are not necessarily part of the secondary cell wall hemicellulose matrix, and that environmental factors may affect their expression patterns.

Thus, xylem differentially expressed genes bring molecular knowledge on key functional and anatomical processes seemingly important for white teak’s secondary xylem development, like the activation of programmed cell death, the activation of biosynthetic pathways related to lignin formation and other components of the secondary cell wall, or other associated regulatory processes.

## Conclusions

Transcriptomic profiling of leaves and wood of young white teak (*G. arborea* Roxb.) trees was carried out, which constitutes an important genomic resource for this tropical timber. Differential expression analysis allowed to identify, for the first time in this species, major genes related with lignin biosynthesis and other components of the secondary cell wall, as well as the main transcription factors implicated in its regulation. Also, a catalog of intragenic microsatellite markers was obtained that may be useful in the future establishment of strategies for marker assisted selection of traits related with lignin formation, wood and/or secondary cell wall development in this economically important tree species. The transcriptome obtained could contribute significantly to increase the knowledge on wood and lignin formation that is still scarce in white teak, and will be highly useful for other non-model tropical wood tree species.

## Methods

### Plant material and RNA isolation

Plant material was obtained from approximately one-year-old trees, located in the commercial plantation “El Neme”, located at Coello (Tolima, Colombia). Leaves and stem cuttings from six different individual plants were collected and stored in liquid nitrogen. For RNA isolation from stem (with secondary xylem), external tissues that constitute the bark (phloem and periderm), and pith were removed from stems. Wood was chopped into small pieces using a sterile scalpel and grounded in liquid nitrogen. Total RNA was obtained using the protocol developed for RNA extraction from the pine wood by Chang et al. [74]. The leaf RNA was isolated using the Isolate I RNA isolation kit (Bioline, BIO-52040). RNA samples were quantified using a Nanodrop spectrophotometer (2000, Thermo Scientific, USA) and its integrity was verified using 1% agarose gel electrophoresis in denaturing conditions.

### Library preparation and RNA-seq

RNA samples with best integrity and concentration values were further validated using a bioanalyzer (2100 Agilent, USA) and samples with a RIN value > 7 were selected for sequencing. Nine RNA samples of each, xylem and leaves, were used to make 3 pools of 3 different individuals for each tissue type. From each pool of RNA, sequencing libraries were generated using the TruSeq library prep kit (Illumina, catalog no. RS-122-210, USA), obtaining six indexed libraries with three replicates for each tissue. All the libraries were sequenced using the NextSeq500 platform (Illumina, USA) to generate paired-end reads of 2 × 150 bases length.

### Bioinformatic analysis and de novo assembly of reference transcriptomes

Raw RNA-seq reads were evaluated for quality, and sequences with a Q score < 20 were eliminated. Adapters were eliminated by trimming the 10 bp from the 5′ ends of the reads using Trimmomatic (version 0.36) [75]. Additionally, the reads corresponding to rRNAs were aligned and eliminated using the program bowtie2 (version 2.3.5) [76] and the SILVA database [77]. Finally, overrepresented sequences identified as contaminants or low complexity sequences were also eliminated from the further processing.

A de novo transcriptome assembly strategy was chosen discarding the alternative of reference genome-guided assembly, because the most closely related genome sequence available belongs to a relatively distant member of the Lamiaceae family, and a different genus (*T. grandis*). Thus, transcriptome assembly was performed using Trinity (version 2.1.1) [78], setting as parameters a minimum length of 200 bases and a k-mer value of 25. To obtain the transcriptome of secondary xylem, only the reads from stem were used in the assembly process. Additionally, filtered reads from xylem (stem) and leaves were pooled and assembled to obtain a combined reference transcriptome for the differential expression analysis. All the necessary softwares for the computational analysis were run using the High-Performance Computational Center (HPCC) at Texas Tech University and the ZINE Cluster of Xavierian University.

### Transcriptome annotation

Transcriptome annotation was performed using the BLASTX similarity search program [79] against different public databases (TAIR10, NR, and UNIPROT/SWISSPROT) and employing an e-value of 1e-5 as cut-off value. Categories of gene ontology (GO) were assigned using the GO annotation tool in TAIR [80]. For visualization of GO categories, the system of classification of Wego was used [81]. TAIR annotation was also used for the identification of transcription factors using the AGRIS transcription factors database [82]. KO identifiers necessary for the annotation in KEGG pathways were obtained using the Uniprot tools [83]. PFAM domains were identified using HMMER tools [84]. Additionally, the TRAPID tool was used to perform a quick annotation based in RAPSearch and identify ORFs in the transcripts [85]. For the validation of the identity of some genes with full length ORFs, a multiple alignment–based phylogenetic analysis of their derived protein sequences was performed with selected homologous sequences of plant model and tree species obtained from gene bank, Uniprot, TAIR, PlantTFDB and iTAK plant transcription factor database, using the MEGA 7 software [86].

### SSRs identification

The xylem reference transcriptome was further analyzed for the presence of microsatellite markers using the MISA tool [87], considering a minimum of 5 motif repetition for the dinucleotides (DNRs), trinucleotides (TNRs), tretranucleotides (TtNRs), pentanucleotides (PNRs) and hexanucleotides (HNRs).

### Differential expression analysis

For differential expression analysis, the transcriptome obtained from the assembly of pooled reads from xylem (stem) and leaf tissues was used as the reference transcriptome. Reads from each replicate and tissue were aligned against this reference transcriptome using bowtie2 and samtools [88] and the counts of the mapped sequences were obtained using bedtools [89]. Counts were normalized to FPKMs (Fragments Per Kilobase per Million mapped reads) and the differential expression analysis was performed using DESeq package [90] with the leaf transcripts used as control tissue. A principal component analysis (PCA) of expression levels and using transcripts counts was performed to assess the variance in transcript profiling simultaneously amongst samples (replicates) and treatments (i.e. tissues: xylem and leaves). PCA plot was obtained using ggplot2 R package [91].

Selection of differentially expressed genes between xylem and leaf tissue was done using a binomial test with an adjusted *p*-value (*p* < 0.05) and values of logarithmic to base 2 of expression fold change (log_2_ FC) ≥ 2 or ≤ − 2, indicating up- or down-regulation of the xylem genes in comparison with leaves. The functional annotation of differentially expressed transcripts was performed using the Mercator [92] and TRAPID [85] tools. Visualization of the key metabolic pathways with differentially expressed genes was performed using the MapMan program [93].

### Differential expression validation of genes using RT-qPCR

To validate the differential expression of a selection of genes upregulated in xylem, RT-qPCR was performed. cDNA of xylem (stem) and leaves were prepared using the Transcriptor first strand cDNA synthesis kit (Roche, USA): 1 μg of total RNA per 40 μl final reaction volume was used following manufacturer operating procedure. Primers were designed for the selected 13 candidate genes related to the monolignol biosynthetic pathway, cellulose and hemicellulose synthesis, and transcription factors involved in the regulation of secondary cell wall biosynthesis. *UBIQUITIN5* (*UBQ5*), *β*-*TUBULIN* (*β-TUB*) and *HISTONE3* (*HIS3*) genes were evaluated as reference genes based on the transcriptome data and finally *UBIQUITIN5* (*UBQ5*) was used for the normalization of RT-qPCR data. Primer3 tool was used for the primer designing taking into account the criteria for qPCR primers [94] (Supplementary Table 3).

RT-qPCR was run in a Lightcycler 96 real time PCR (Roche, USA) using the Fast start™ SYBR green (Roche, USA in a 96-well plate with 3 biological replicates and 3 technical replicates for each gene. Reactions were performed by manufacturer operating procedure in a final volume of 20 μl with 10 μl of SYBR mix, 5 μl of five-fold diluted cDNA (equivalent to 25 ng of reverse transcribed total RNA) and primers at a final concentration of 0.5 pmol/μl. Three negative template controls per primer pair were included in each plate. Running conditions were: a pre incubation phase at 95 °C for 10 min, 45 cycles of amplification with 3 steps: 95 °C for 10 s, 58 °C for 10 s and 72 °C for 10 s, a melting phase with 3 steps: 95 °C for 10 s, 65 °C for 60 s and 97 °C for 1 s, finally a cooling phase at 37 °C for 30 s. Melting curves were analyzed to verify the presence of only one product and the absence of primer dimers. The ΔΔCt comparative method [95] was used for the estimation of the change of gene expression between the two tissues.

## Supplementary Information

**Additional file 1: Supplementary Fig. 1.** Principal component analysis (PCA) of *G. arborea* expressed transcripts. Transcript read counts obtained in each sample were used. Difference between plant tissues (condition) is highlighted.

**Additional file 2: Supplementary Fig. 2**. Main functional categories represented by DEG.

**Additional file 3: Supplementary Table 1**. Summary of *G. arborea* de novo transcriptome assembly metrics combining RNA-seq data from leaves and xylem.

**Additional file 4: Supplementary Table 2.** Frequency in the number of repetitions found for SSRs microsatellite markers.

**Additional file 5: Supplementary Table 3.** Primers used for RT-qPCR validation of differentially expressed genes between xylem and leaf tissues.

## Data Availability

Sequences for comparative phylogenetic analysis were downloaded from Uniprot (https://www.uniprot.org/), NCBI protein (https://www.ncbi.nlm.nih.gov/protein/), TAIR (https://www.arabidopsis.org), PlantTFDB (http://planttfdb.gao-lab.org/) or iTAK (http://itak.feilab.net/cgi-bin/itak/index.cgi) databases. Accession numbers of all downloaded sequences employed for multiple alignments and phylogenetic analyses are listed in Fig. 3 and Fig. 9 legends. All assembled sequences of this transcriptomic resource have been deposited in the European Nucleotide Archives (ENA) public database under the following accession numbers: PRJEB36634 (ERP119847). The data supporting the conclusions of this article are included within the article and its supplementary information files.

## Abbreviations

CAD: cinnamyl alcohol dehydrogenase
CRISPR-CAS: Clustered Regularly Interspaced Short Palindromic Repeats- CRISPR Associated Nuclease
GO: gene ontology
PAL: Phenylalanine ammonia-lyase
C4H: *trans*-cinnamate 4-hydroxylase
4CL: 4-coumarate-CoA ligase
HCT: *p*-hydroxy-cinnamoyl-CoA
CCoAOMT: caffeoyl-CoA *O*-methyltransferase
CCR: hydroxycinnamoyl-CoA reductase
F5H: ferulate 5-hydroxylase
COMT: caffeic acid *O*-methyltransferase
SSR: Single Sequence Repeat
KEGG: Kyoto encyclopedia of genes and genomes
ORF: open reading frame
FPKMs: Fragments per kilobase per million mapped reads
PCA: principal component analysis
Log_2_FC: log_2_ (fold change)
DEGs: Differentially expressed genes
RT-qPCR: quantitative reverse transcription PCR
IRX: Irregular xylem
CesA: cellulose synthase
PGSIP: plant glycogenin-like starch initiation proteins
CSL: cellulose synthase-like protein
