# Supplementary figures and images for: De novo transcriptome analysis of white teak (Gmelina arborea Roxb) wood reveals critical genes involved in xylem development and secondary metabolism
Source: BMC Genomics. 2021 Jul 2;22:494. doi: 10.1186/s12864-021-07777-x (PMC8252223; doi:10.1186/s12864-021-07777-x)

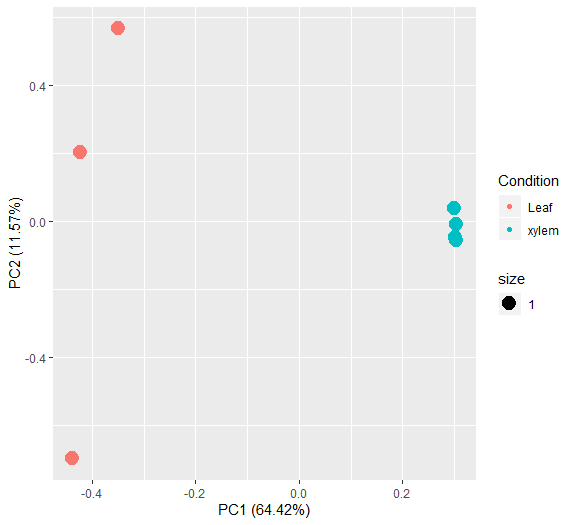

Supplement: Supplementary file 1 — Additional file 1: Supplementary Fig. 1. Principal component analysis (PCA) of G. arborea expressed transcripts. Transcript read counts obtained in each sample were used. Difference between plant tissues (condition) is highlighted. [file 12864_2021_7777_MOESM1_ESM.png]

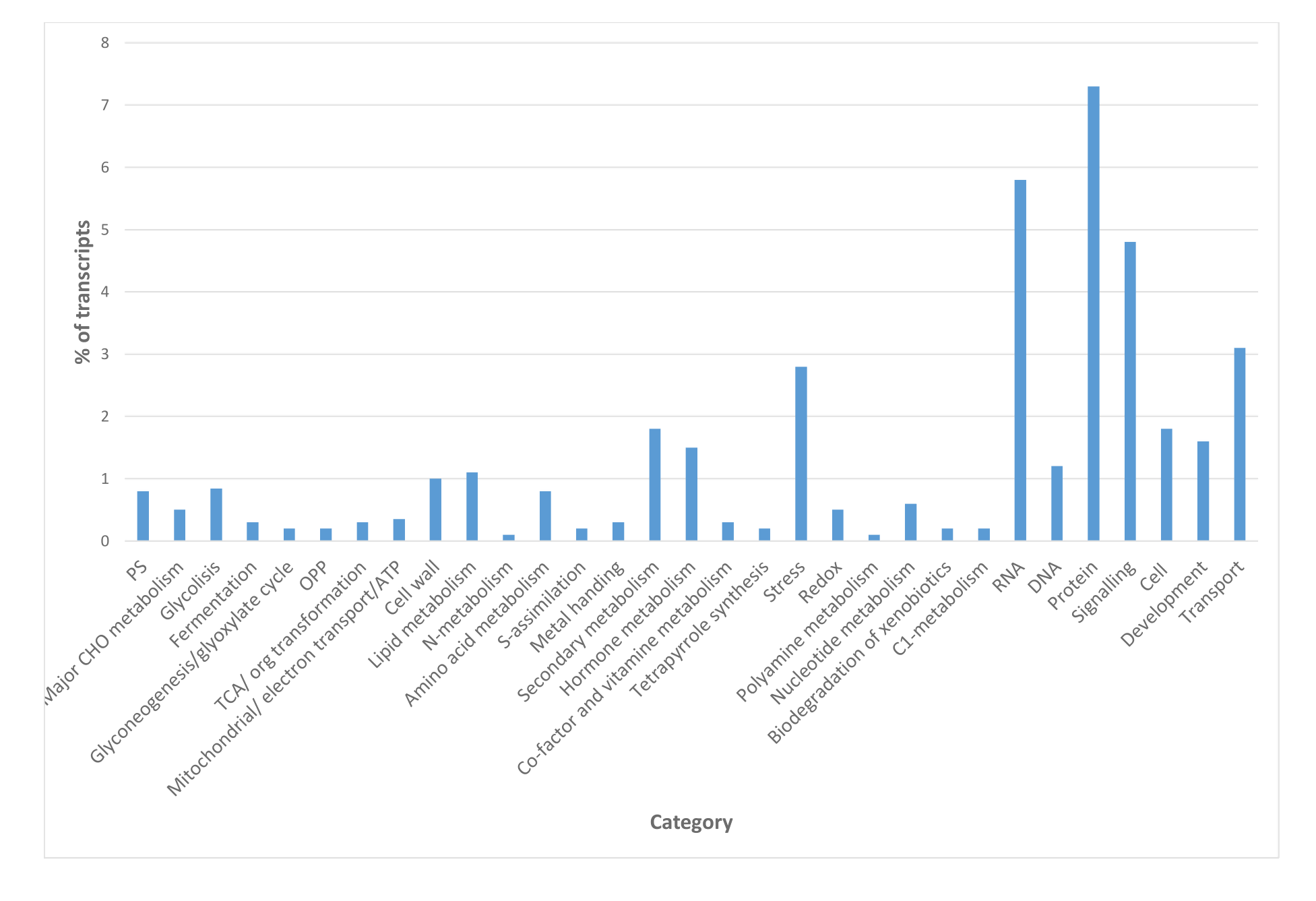

Supplement: Supplementary file 2 — Additional file 2: Supplementary Fig. 2. Main functional categories represented by DEG. [file 12864_2021_7777_MOESM2_ESM.png]
