# Supplementary material for: De novo transcriptome analysis of white teak (Gmelina arborea Roxb) wood reveals critical genes involved in xylem development and secondary metabolism
Source: BMC Genomics. 2021 Jul 2;22:494. doi: 10.1186/s12864-021-07777-x (PMC8252223; doi:10.1186/s12864-021-07777-x)
Supplement: Supplementary file 3 — Additional file 3: Supplementary Table 1. Summary of G. arborea de novo transcriptome assembly metrics combining RNA-seq data from leaves and xylem. [file 12864_2021_7777_MOESM3_ESM.docx]

**Supplementary table 1**. Summary of *G. arborea* de novo transcriptome assembly metrics combining RNA-seq data from leaves and xylem.

| **Assembly** | |
| --- | --- |
| Total number of leaf reads (filtered) | 147,130,884 |
| Total number of xylem reads (filtered) | 164,718,354 |
| Number of reads used for the assembly | 311,849,238 |
| Number of transcripts obtained post assembly | 151,229 |
| **N50** value (in nt) | 1332 |
| Average contig length (in nt) | 782.84 |
| Number of bases assembled | 118,457,690 |
| **Annotation** | |
| Full length ORFs | 20,156 |
| Quasi full length ORFs | 16,706 |
| Transcripts with hits in TAIR10 (blastx) | 53,537 |
| Transcripts with Interpro domains | 47,884 |
| Transcripts classified in gene families | 57,075 |
| Transcripts with GO terms | 41,674 |
| Number of GO terms | 3106 |
