# Supplementary material for: De novo transcriptome analysis of white teak (Gmelina arborea Roxb) wood reveals critical genes involved in xylem development and secondary metabolism
Source: BMC Genomics. 2021 Jul 2;22:494. doi: 10.1186/s12864-021-07777-x (PMC8252223; doi:10.1186/s12864-021-07777-x)
Supplement: Supplementary file 4 — Additional file 4: Supplementary Table 2. Frequency in the number of repetitions found for SSRs microsatellite markers. [file 12864_2021_7777_MOESM4_ESM.docx]

**Supplementary table 2.** Frequency in the number of repetitions found for SSRs microsatellite markers.

| **Unity Size** | **Number of SSRs** |
| --- | --- |
| 2 | 20,634 |
| 3 | 4463 |
| 4 | 319 |
| 5 | 17 |
| 6 | 27 |
