# Supplementary material for: De novo transcriptome analysis of white teak (Gmelina arborea Roxb) wood reveals critical genes involved in xylem development and secondary metabolism
Source: BMC Genomics. 2021 Jul 2;22:494. doi: 10.1186/s12864-021-07777-x (PMC8252223; doi:10.1186/s12864-021-07777-x)
Supplement: Supplementary file 5 — Additional file 5: Supplementary Table 3. Primers used for RT-qPCR validation of differentially expressed genes between xylem and leaf tissues. [file 12864_2021_7777_MOESM5_ESM.docx]

**Supplementary table 3.** Primers used for RT-qPCR validation of differentially expressed genes between xylem and leaf tissues.

| Gene /Primer | Sequence 5´-3´ | Amplicon Size |
| --- | --- | --- |
| *PAL-F* | AAGGCATTGCATGGAGGGAA | 201 |
| *PAL-R* | CTCAGCACCCTTGAACCCAT |  |
| *4CL-R* | TTGACGGTGATGACGAGCTC | 209 |
| *4CL-F* | CTCAGTGACAGCGGAACCAT |  |
| *CADx-F* | GACTCAACAAACCTGGTATGCACA | 392 |
| *CADx-R* | CGTCTCTTTCATCCCTCCAATGC |  |
| *CADl-R* | GATAGGCACAATGGATGGTATCG | 171 |
| *CADl-F* | GCGCTTCCAACTATCGCCC |  |
| *COMT-F* | GACAGGGTCTTGATGGAGGC | 232 |
| *COMT-R* | CACCACCAACATCGACCAGA |  |
| *CCoAOMT-F* | GCATCAGGAGGTTGGGCATA | 238 |
| *CCoAOMT-R* | AGAGTAGCCGGTGTAAACGC |  |
| *CCR-F* | CTGGAACAGTGATGGGTCCT | 232 |
| *CCR-R* | GCCACCTTAGCAGCAAAATC |  |
| *HCT-F* | CTTTGTGTGGCGAACTCGTA | 229 |
| *HCT-R* | TTACCCCATCCAAAATCTGC |  |
| *NST1-F* | ATGGCCAGAAATCAGACTGG |  |
| *NST1-R* | ATGAAGTGAGGGGGCTTTCT | 177 |
| *MYB85-F* | AGCTGCCTATTCAGGGATGA |  |
| *MYB85-R* | TGTCGCTGAAACAATCGAAG |  |
| *FRA8-F* | TGGCTGGCTGACTTTTTCTT | 213 |
| *FRA8-R* | ATCCTCCATGGTGTGAAAGC |  |
| *PGSIP3-R* | CAGCTCACCGACTACGACAA | 230 |
| *PGSIP3-F* | TCGTTCAGAAAACCCTGGTC |  |
| *Ces-R* | TTCCGAAGGCAAGCTCTTTA | 169 |
| *Ces-F* | AGGCATCTCTGTGCTTCGAT |  |
| *UBQ5-F* | GATAGAGGTGGTGCTGAACGA | 179 |
| *UBQ5-R* | AGTCCTTGAGGGTGATGTGG |  |
| *HIST3-F* | GTTGCCTTGAGGGAGATCAG | 176 |
| *HIST3-R* | TCTTAGCGTGAATCGCACAC |  |
| *βTUB-F* | TGGTGATCTCAACCACCTCA | 211 |
| *βTUB-R* | GATACTGCTGGGAGCCTCTG |  |
